# Structural rationalities of tapered hollow cylindrical beams and their use in Japanese traditional bamboo fishing rods

**DOI:** 10.1038/s41598-022-06426-x

**Published:** 2022-02-14

**Authors:** Ryo Nishiyama, Motohiro Sato

**Affiliations:** 1 Division of Mechanical and Space Engineering, Graduate School of Engineering, Hokkaido University, N-13, W-8, Kita-ku, Sapporo, 060-8628 Japan; 2 Division of Mechanical and Aerospace Engineering, Faculty of Engineering, Hokkaido University, N-13, W-8, Kita-ku, Sapporo, 060-8628 Japan

**Keywords:** Engineering, Mechanical engineering

## Abstract

Bamboo has historically been used in Japan as a structural material and for building tools such as fishing rods owing to its remarkable structural properties. In recent years, the materials used for manufacturing fishing rods have changed greatly owing to the development of composite materials; however, the basic slender tapered hollow cylindrical fishing rod design has remained unchanged throughout the long history of fishing. However, the mechanical rationale behind this structural design has not yet been sufficiently verified, and this study clarifies this. The analysis was performed by solving the nonlinear bending equation of a slender tapered cantilever beam with a concentrated load at the tip, which causes large deflection, using the Runge–Kutta method. The deflection curves and bending stresses were obtained, and the structural design to minimize the stresses was explored. Our results may prove useful for bamboo-inspired bionic design and bring to light our ancestors’ deep knowledge of natural materials and their advanced technological capabilities.

## Introduction

The bamboo plant^1^ flourishes in warm and humid regions of Asia and has been widely used as a structural material and for making tools since ancient times owing to its flexibility and strength^2,3^. Bamboo acquired its unique traits because it had to adapt to its harsh natural environment. Understanding the mechanical rationality of bamboo will aid the design of an ideal biomimetic hollow cylindrical structure that simultaneously provides high strength and high rigidity with efficient use of materials. Therefore, bamboo-inspired bionic design has been heavily researched in recent years^4–14^.

Amada and Nagase^15^ focused on the characteristics of flexure and estimated the wind load applied to the trunk by the branches and leaves and their own weight from the projected area of the photographs, and conducted a large deflection analysis based on the measured data of bamboo. They confirmed that the bamboo effectively suppressed the stress increase by shifting the position of maximum bending stress generation toward the root, which is more rigid, by bending the whole trunk at high wind loads. This demonstrates an example of the mechanical advantages of the structural properties of flexure.

**Figure 1:**
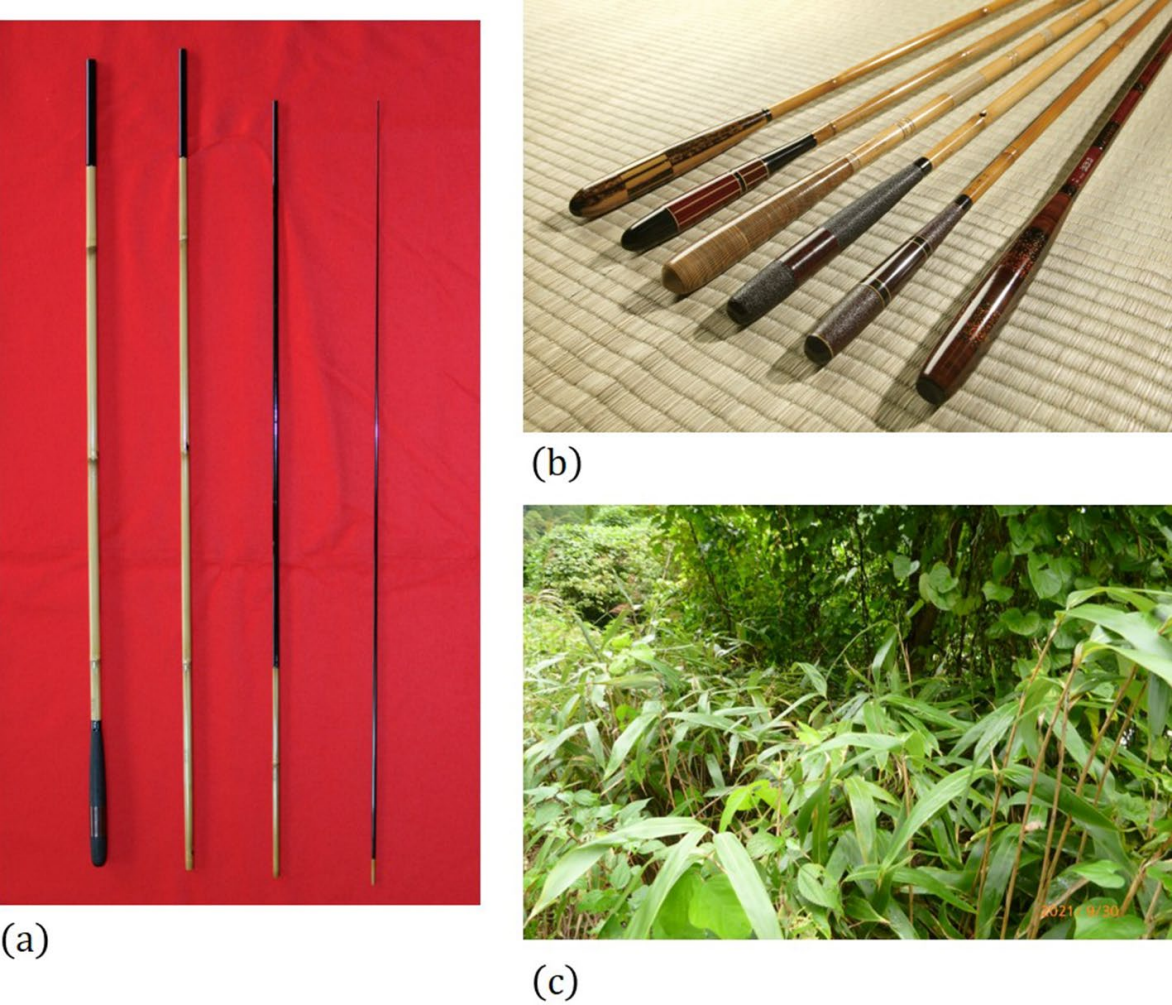
(**a**,**b**) Photographs of *kisyuu-herazao*, a type of Japanese bamboo rod, which has a history exceeding 100 years and is designated as one of Japan's traditional crafts (Courtesy of Hashimoto-City, Wakayama, Japan), (**c**) Bamboo grove of species *Sasamorpha borealis*, which is used to make the *kisyuu-herazao* because of its hardness and rigidity (Courtesy of Hashimoto-City, Wakayama, Japan).

A familiar example of utilizing the flexure of such a slender rod is the fishing rod. In recent years, composite materials such as carbon fiber-reinforced plastic (CFRP) and glass fiber-reinforced plastic (GFRP) have made remarkable progress, and they are used in fishing rods. However, the basic slender tapered rod design of the fishing rod has remained unchanged throughout the long history of fishing. Turning now to the history of fishing, the origins of the fishing rod is bamboo rods (Fig. 1). This is probably because bamboo provides a naturally slender tapered hollow cylindrical structure, such a structure provides useful properties for fishing. The study of the large deflection of bamboo and fishing rods ultimately leads to the nonlinear large deformation problem of slender tapered rods^16–21^. Similar large-deformation behaviors are important in medical, sports, and industrial applications^22–25^. Therefore, a theoretical understanding of this phenomenon can be useful in a large number of academic fields.

Matsumura and Jimbo^26^ extensively investigated the mechanical properties and behavior of slender tapered rods from the perspective of applied-mechanics, but the evaluation is based on the dimensionless load divided by the taper ratio, and the discussion is limited to the comparison between rods with the same taper ratio. Therefore, this study optimized the shape of rods by deriving the optimal taper that minimizes the bending stress under the equal volume and height conditions. In addition, by considering the hollowing of the rod, the mechanical rationality behind the widespread use of bamboo in fishing rods in Japan were discussed.

## Results

### Derivation of basic equations

**Figure 2:**
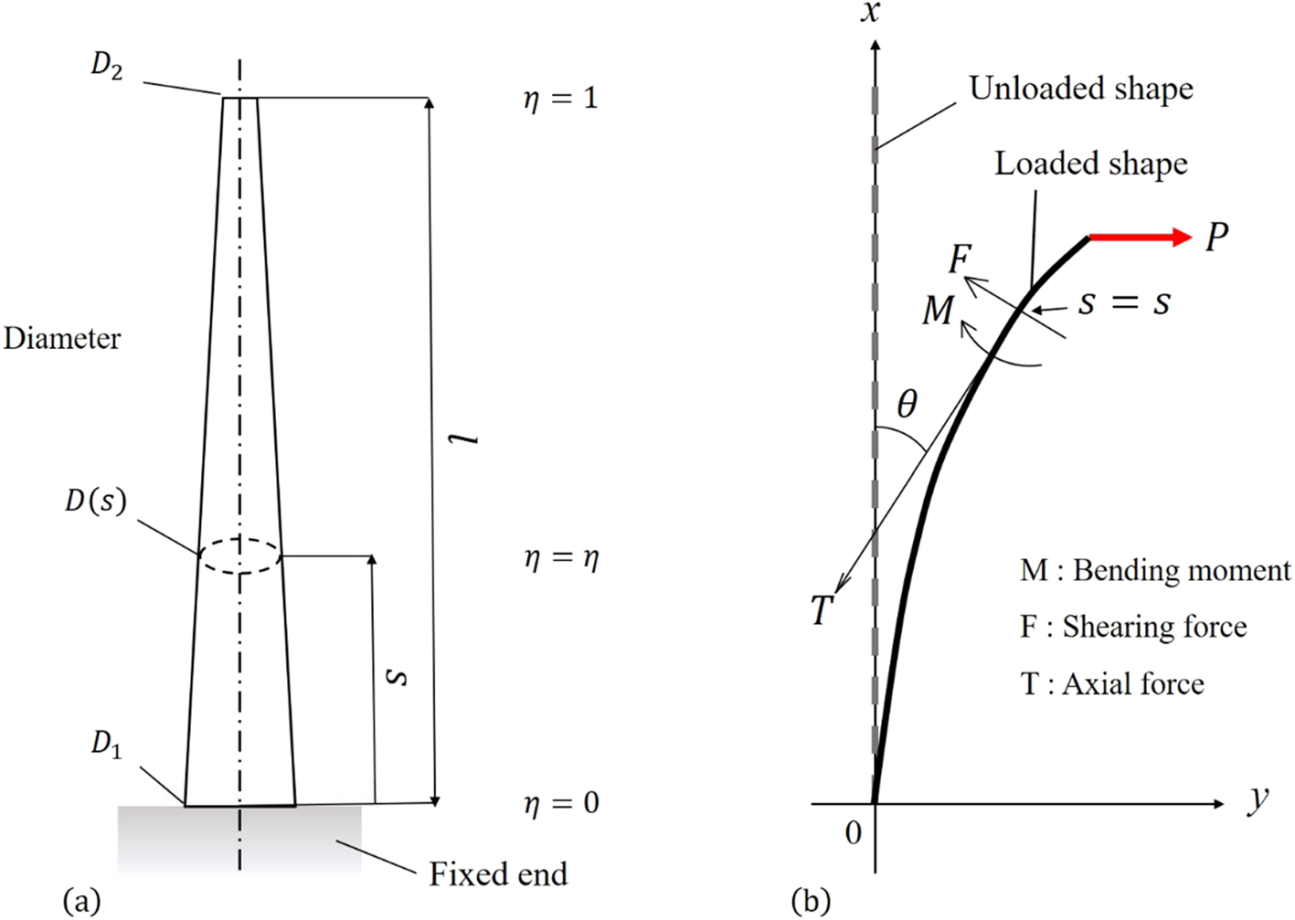
(**a**) Slender tapered rod with a circular cross section, (**b**) Deflection angle at any relative height of a cantilever beam under concentrated load.

In this study, a slender tapered rod of length $l$ is considered as a cantilever beam with a fixed end at the large end, as shown in Fig. 2a. The intersection point of the neutral axis and fixed end is designated the origin; the fixed end of the neutral axis is defined at $s = 0$ and the free end at $s = l$. The free end is subject to a concentrated load $P$ in the horizontal direction, and the member is assumed to be an isotropic elastic body with a constant longitudinal modulus of elasticity $E$. The cross-sectional forces acting on an arbitrary cross-section are the axial force $T$ (positive for tension), shear force $F$, and bending moment $M$ (Fig. 2b).

The cross-sectional diameters of the rod at the fixed and free ends are $D_{1}$ and $D_{2}$, respectively. $D_{1}$ is assumed to be proportional to $D_{2}$, and the proportionality constant is called the taper ratio, denoted by $\xi$ ($= D_{2} /D_{1}$). In this study, the diameter $D\left( s \right)$ of the model is expressed by Eq. (1).

1 $$ D\left( s \right) = D_{1} \left\{ {1 - \left( {1 - \xi } \right)\frac{s}{l}} \right\} $$

The cross-sectional area $A\left( s \right)$ and cross-sectional second moment $I\left( s \right)$ are functions of $s$, and the diameter varies as a linear function of $s$. The following Eqs. (2) and (3) are obtained.

2 $$ A\left( s \right) = A_{1} \left\{ {1 - \left( {1 - \xi } \right)\frac{s}{l}} \right\}^{2} , A_{1} = \frac{{\pi D_{1}^{2} }}{4} $$

3 $$ I\left( s \right) = I_{1} \left\{ {1 - \left( {1 - \xi } \right)\frac{s}{l}} \right\}^{4} , I_{1} = \frac{{\pi D_{1}^{4} }}{64} $$

The volume $V_{12}$ of the slender tapered rod can be expressed by Eq. (4). Therefore, if this tapered rod is converted into an untapered rod under the same volume and height conditions, its diameter $D_{{\text{o}}}$ can be expressed by Eq. (5) using $D_{1}$.

4 $$ V_{12} = \mathop \smallint \limits_{0}^{l} A\left( s \right)ds = \frac{{\pi D_{1}^{2} l}}{12}\left\{ {1 + \xi + \xi^{2} } \right\} $$

5 $$ D_{{\text{o}}} = D_{1} \sqrt {\frac{{1 + \xi + \xi^{2} }}{3}} $$

The theoretical analysis is conducted in the elastic region and the members are assumed to be inextensible; thus, the Euler–Bernoulli assumption is valid. The effects of shear force and the Poisson effect are neglected. The basic equation for bending is given by Eq. (6). Differentiating Eq. (6) with respect to $s$, we obtain Eq. (7).

6 $$ E\left\{ {1 - \left( {1 - \xi } \right)\frac{s}{l}} \right\}^{4} I_{1} \frac{d\theta }{{ds}} = M $$

7 $$ \left\{ {1 - \left( {1 - \xi } \right)\frac{s}{l}} \right\}^{4} \frac{{d^{2} \theta }}{{ds^{2} }} - \frac{4}{l}\left( {1 - \xi } \right)\left\{ {1 - \left( {1 - \xi } \right)\frac{s}{l}} \right\}^{3} \frac{d\theta }{{ds}} = - \frac{P\cos \theta }{{EI_{1} }} $$

The ratio $s/l$ is defined as $\eta$. Furthermore, if the cross-sectional second moment of the untapered rod is $I_{{\text{o}}}$ and the dimensionless load is $\alpha_{{\text{o}}} = Pl^{2} /EI_{{\text{o}}}$, the second-order nonlinear differential equation can be obtained as shown in Eq. (8). In this study, the deflection due to the weight of the rod is assumed to be smaller than that due to the concentrated load $P$, and the effect of its own weight is ignored.

8 $$ \left\{ {1 - \left( {1 - \xi } \right)\eta } \right\}^{4} \frac{{d^{2} \theta }}{{d\eta^{2} }} - 4\left( {1 - \xi } \right)\left\{ {1 - \left( {1 - \xi } \right)\eta } \right\}^{3} \frac{d\theta }{{d\eta }} + \frac{{\left( {1 + \xi + \xi^{2} } \right)^{2} }}{9}\alpha_{{\text{o}}} \cos \theta = 0 $$

Equation (8) was solved by direct numerical analysis using the Runge–Kutta method. From the analysis, the deflection angle $\theta \left( \eta \right)$ and curvature $d\theta \left( \eta \right)/d\eta$ at point $\eta$ were determined, and the large deflection curve and bending stress were derived as described below. The coordinates $\left( {x/l, y/l} \right)$ at point $\eta$ are expressed by Eq. (9), and the dimensionless bending stress $l\sigma_{{\text{M}}} /ED_{{\text{o}}}$ is expressed by Eq. (10).

9 $$ \left( {\frac{x}{l}, \frac{y}{l}} \right) = \left( {\mathop \smallint \limits_{0}^{\eta } \cos \theta d\eta , \mathop \smallint \limits_{0}^{\eta } \sin \theta d\eta } \right) $$

10 $$ \frac{{l\sigma_{{\text{M}}} }}{{ED_{{\text{o}}} }} = \frac{l}{{ED_{{\text{o}}} }}\frac{M}{I}\frac{D}{2} = \frac{1}{2}\left\{ {1 - \left( {1 - \xi } \right)\eta } \right\}\sqrt {\frac{3}{{1 + \xi + \xi^{2} }}} \frac{d\theta }{{d\eta }} $$

### Large deflection deformation

**Figure 3:**
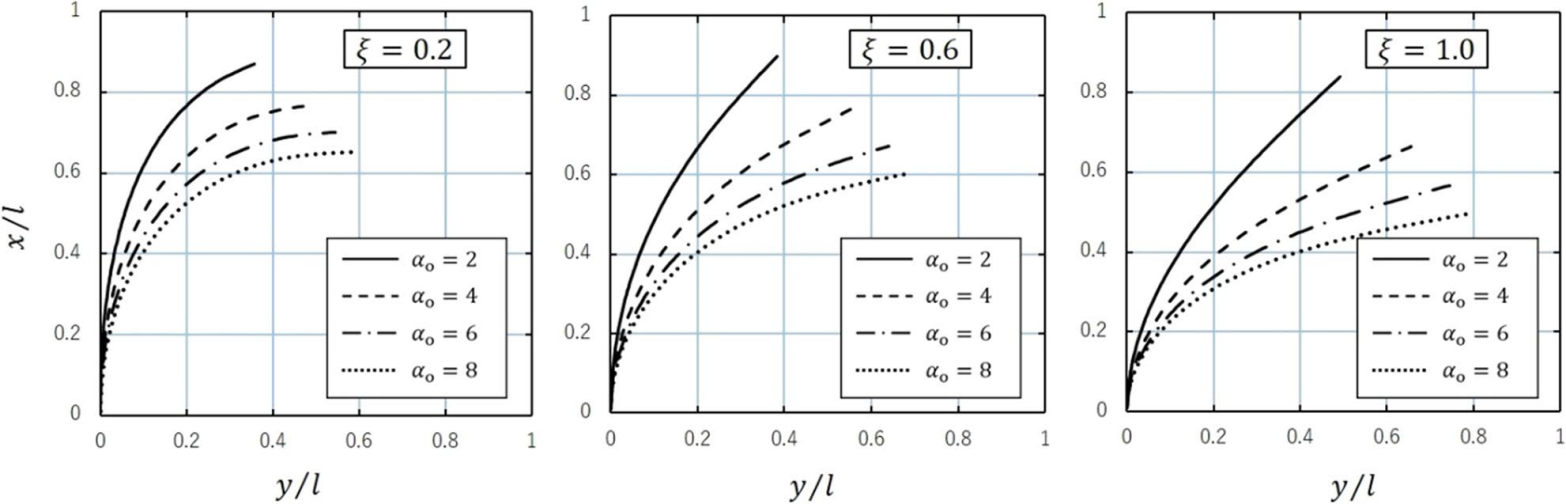
Deflection curves for $\xi = 0.2, 0.6, \;{\text{and}}\; 1.0$ and $\alpha_{{\text{o}}} = 2, 4, 6,\; {\text{and}}\;{ }8$.

Figure 3 illustrates the aspects of large deflection deformation when the dimensionless load $\alpha_{{\text{o}}}$ is varied between 2, 4, 6, and 8, with taper ratios $\xi$ of 0.2, 0.6, and 1.0. The dimensionless load $\alpha_{{\text{o}}}$ used in this study is based on the values used in previous studies^20,21,26^. When the taper ratio was large and the shape was close to a cylinder, the rod deflected greatly from the root, while the deformation behavior changed as the diameter ratio decreased and the shape approached a cone, bending considerably near the tip. Ohtsuki and Takeuchi^21^ showed that the analytical large deformation behaviors of tapered rods are in agreement with the experimental solutions.

**Figure 4:**
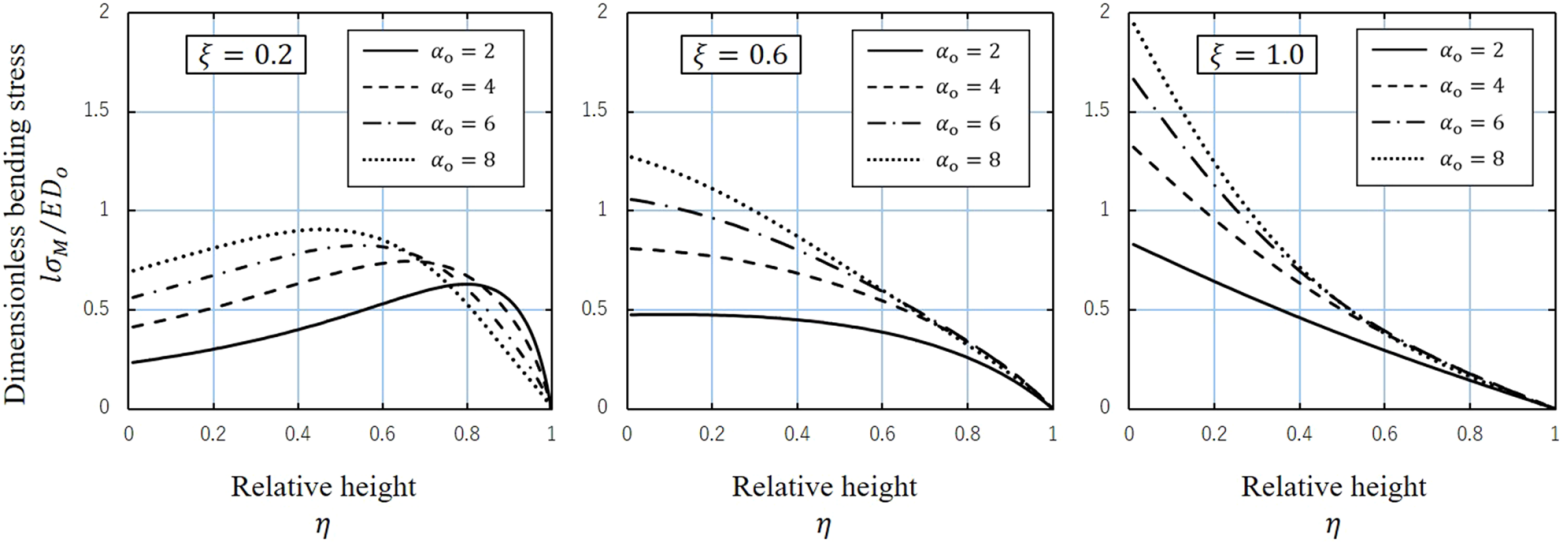
Variation of the dimensionless bending stress $l\sigma_{{\text{M}}} /ED_{{\text{o}}}$ with the relative height $\eta$ for $\xi = 0.2, 0.6,\;{\text{and}}\; 1.0$ and $\alpha_{{\text{o}}} = 2, 4, 6, \;{\text{and}}\; 8$.

Figure 4 illustrates the bending stress $l\sigma_{{\text{M}}} /ED_{{\text{o}}}$ versus relative height $\eta$ curve obtained by varying the taper ratio $\xi$ between 0.2, 0.6, and 1.0 and the dimensionless load $\alpha_{{\text{o}}}$ between 2, 4, 6, and 8. When the taper ratio is large and the shape is close to cylindrical, the stress decreases monotonically with increasing height. However, when the taper ratio is small and the shape is close to conical, the stress tends to increase and then decrease, and as $\alpha_{{\text{o}}}$ increases, the maximum value moves toward the large end where the stiffness is large. This extreme value shift is the result of flexure, which effectively suppresses the increase in bending stress in response to an increased load.

### Shape optimization of tapered solid rods

**Figure 5:**
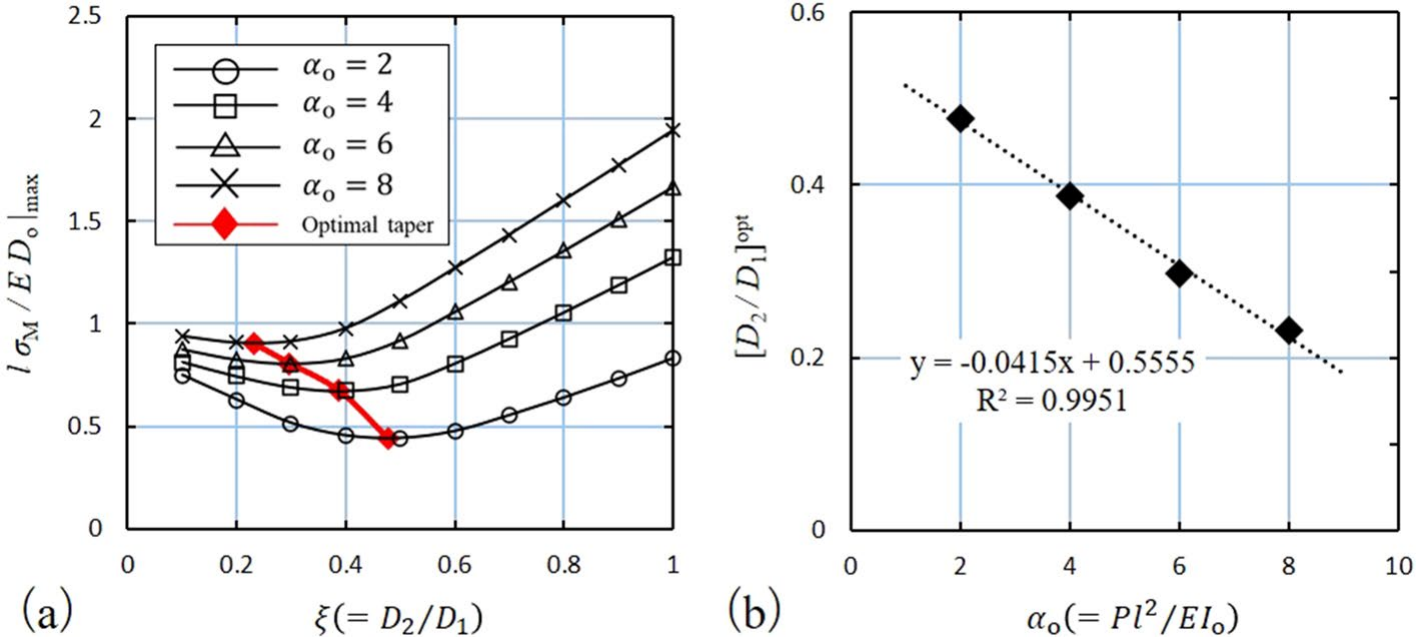
(**a**) Variation of the maximum bending stress $\left. {l\sigma_{{\text{M}}} /ED_{{\text{o}}} } \right|_{{{\text{max}}}}$ with respect to taper $\xi$ for $\alpha_{{\text{o}}} = 2, 4, 6,{\text{ or}} 8$, (**b**) Variation of the optimal taper $\left[ {D_{2} /D_{1} } \right]^{{{\text{opt}}}}$ with respect to $\alpha_{{\text{o}}}$.

Figure 5a shows the maximum value of the dimensionless bending stress $\left. {l\sigma_{{\text{M}}} /ED_{{\text{o}}} } \right|_{{{\text{max}}}}$ for each taper ratio obtained by varying the dimensionless load $\alpha_{{\text{o}}}$ as 2, 4, 6, and 8. The analysis yielded a taper ratio with the minimum $\left. {l\sigma_{{\text{M}}} /ED_{{\text{o}}} } \right|_{{{\text{max}}}}$ corresponding to $\alpha_{{\text{o}}}$. We refer to this taper ratio as the optimal taper, $\left[ {D_{2} /D_{1} } \right]^{{{\text{opt}}}}$. Furthermore, $\left[ {D_{2} /D_{1} } \right]^{{{\text{opt}}}}$ is proportional to $\alpha_{{\text{o}}}$, and Eq. (11) is derived using the linear approximation with the least-squares method (Fig. 5b).

11 $$ \left[ {\frac{{D_{2} }}{{D_{1} }}} \right]^{{{\text{opt}}}} = - 0.0415\alpha_{{\text{o}}} + 0.5555 $$

### Shape optimization of tapered hollow rods

In this section, the mechanical rationality of slender tapered hollow rods is examined from the viewpoint of the optimal taper mentioned above. Let $D^{\prime}\left( s \right)$ be the outer diameter of a tapered solid rod when it is transformed into a tapered hollow rod under equal volume and height conditions. The ratio $\beta \left( { = t/D^{\prime}} \right)$ of the wall thickness $t\left( s \right)$ to the outer diameter $D^{\prime}\left( s \right)$ is constant $\left( {0 < \beta \le 0.5} \right)$, and the outer diameter of the large end $D_{1}^{^{\prime}}$ is expressed by Eq. (12).

12 $$ D_{1}^{^{\prime}} = \frac{{D_{1} }}{{\sqrt {1 - \left( {1 - 2\beta } \right)^{2} } }} = \frac{{D_{{\text{o}}} }}{{\sqrt {1 - \left( {1 - 2\beta } \right)^{2} } }}\sqrt {\frac{3}{{1 + \xi + \xi^{2} }}} $$

**Figure 6:**
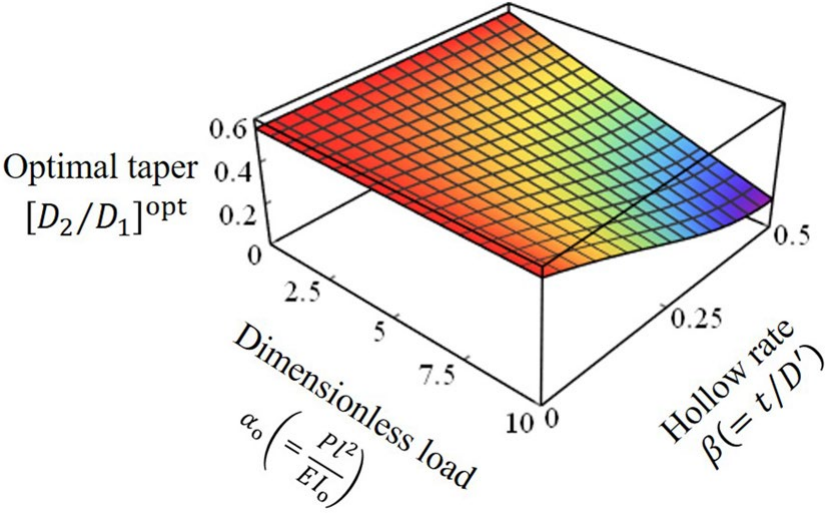
Variation of the optimal taper with the dimensionless load $\alpha_{{\text{o}}}$ and hollow rate $\beta$.

Transforming the right-hand side of Eq. (8) to obtain Eq. (12) gives a nonlinear differential equation (Eq. 13). Therefore, taking the effect of the hollow into account, we can derive an expression for the optimal taper (Eq. 14). Figure 6 shows a 3D plot of the optimal taper of a tapered hollow rod from Eq. (14). The smaller $\beta$ and the more hollow it is, the smaller the variation of the optimal taper $\left[ {D_{2} /D_{1} } \right]^{{{\text{opt}}}}$ with load change. In other words, the hollow structure exhibits mechanically superior structural properties in that it minimizes the bending stress equally over a wide range of loading conditions.

13 $$ \left\{ {1 - \left( {1 - \xi } \right)\eta } \right\}^{4} \frac{{d^{2} \theta }}{{d\eta^{2} }} - 4\left( {1 - \xi } \right)\left\{ {1 - \left( {1 - \xi } \right)\eta } \right\}^{3} \frac{d\theta }{{d\eta }} + \frac{{1 - \left( {1 - 2\beta } \right)^{2} }}{{1 + \left( {1 - 2\beta } \right)^{2} }}\frac{{\left( {1 + \xi + \xi^{2} } \right)^{2} }}{9}\alpha_{{\text{o}}} \cos \theta = 0 $$

14 $$ \left[ {\frac{{D_{2} }}{{D_{1} }}} \right]^{{{\text{opt}}}} = - 0.0415\frac{{1 - \left( {1 - 2\beta } \right)^{2} }}{{1 + \left( {1 - 2\beta } \right)^{2} }}\alpha_{{\text{o}}} + 0.5555 $$

## Mechanical properties of bamboo and their use in fishing rods

Surrounded by the sea on all sides, Japan has one of the world's most prosperous fishing cultures. Bamboo is abundant, light, strong, rigid, and easy to work and meets all the requirements of materials for fishing rods. It is needless to say that the high affinity of bamboo as a material for fishing rods blossomed as a popular culture in the Edo period and played a role in the unique development of bamboo rods up to the present day (Fig. 1).

Present-day fishing rods are typically made of CFRP and GFRP, however, bamboo rods are still treasured, and the techniques have been handed down from generation to generation. Of course, in terms of "unbreakable", bamboo rods will never surpass CFRP or GFRP fishing rods. So why have bamboo rods been loved for so long?

This is because there is an affinity between bamboo and fishing as a structure that goes beyond the material. As shown in this study, the tapered shape of the slender rod effectively suppresses the increase in bending stress with increasing load, and the hollow structure provides not only their own weight reduction but also flexible response to a wide range of loading conditions. In addition, the thinner the hollow structure, the more likely it is to be affected by geometric nonlinearity and cause local buckling. However, actual bamboo has a relatively thick-walled cylindrical structure with a hollow ratio of approximately 0.1, although there are some differences among species and individuals^27^. As a result of repeated self-optimization over a long period of time, bamboo has acquired an exquisite structural balance that is ideal for slender tapered hollow rods with highly refined strength and weight, and can be applied to fishing rods for various fish species and fields.

To reveal and utilize the inherent mechanical rationality of living things is no mean feat even with modern technology, and we have great respect for the deep knowledge of natural materials and advanced technology of our ancestors that made this possible.

## Method

Equation (8) is a nonlinear differential equation with $\eta$ as a variable and is difficult to solve analytically. Therefore, the Runge–Kutta method was used to obtain a direct numerical solution, as in previous studies^20,21,26^. The numerical program Mathematica was used for the analysis.

**Figure 7:**
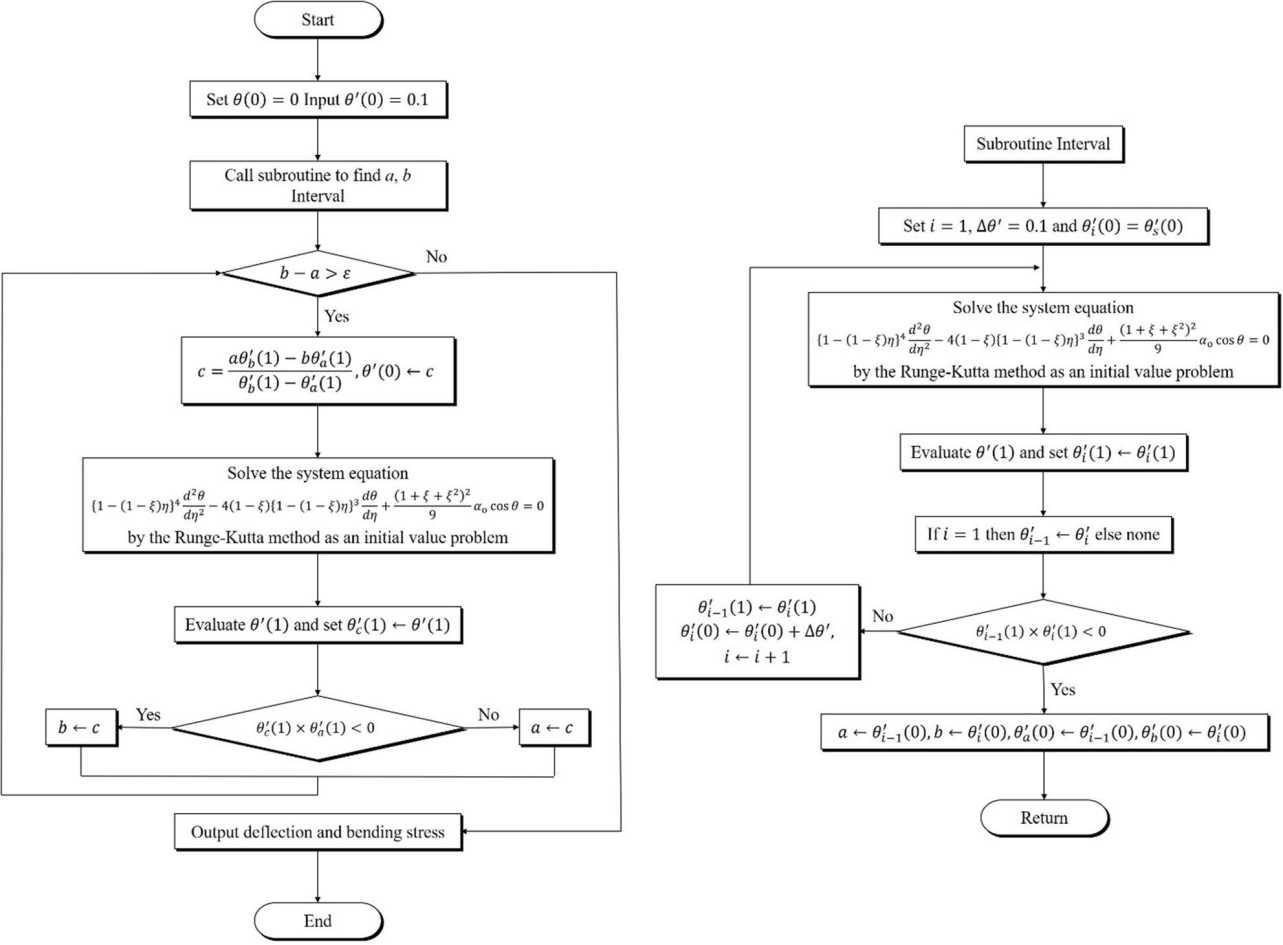
Numerical analysis flow chart of the large deflection using the Runge–Kutta method^20^.

The calculation method is described as follows. Let the initial conditions be $\theta \left( 0 \right) = 0$ and $d\theta \left( 0 \right)/d\eta = \theta_{{\text{o}}}^{^{\prime}}$ where $\theta_{{\text{o}}}^{^{\prime}}$ is the unknown curvature of the neutral axis at the large end. The initial value $\theta_{{\text{o}}}^{^{\prime}}$ was chosen arbitrarily, and the calculation was performed up to the free end ($\eta = 1$). The calculation was iterated until the value of $\theta_{1}^{^{\prime}}$ at the free end was less than the allowable error ($\varepsilon = 10^{ - 10}$). If $\theta_{1}^{^{\prime}}$ did not converge, the initial value of $\theta_{{\text{o}}}^{^{\prime}}$ was modified using the pincer method^28^. The entire algorithm for obtaining the solution is summarized in the flowchart shown in Fig. 7.

## Conclusions

This study revealed the mechanical advantages of bamboo rods from the viewpoint of material mechanics. The findings of this study are summarized as follows.

1. The shape of the deflection curve and bending stress curve depends on the taper ratio and dimensionless load.
2. Slender tapered rods effectively suppress the stress increase by moving the position of maximum bending stress generation toward the root, which has higher rigidity, in response to an increase in external force.
3. The shape of the slender rod was optimized by changing the taper and dimensionless load under equal volume and height conditions. In addition, considering the hollowing of the rods, the optimal taper was derived from the dimensionless load and hollowing ratio.
4. It was shown that the bending stress could be minimized equally for various ranges of loading conditions by hollowing out slender tapered rods.
5. In the regions where bamboo flourished, the origin of the fishing rod was bamboo rods. This is because there is an affinity between the two as a structure that goes beyond the material.

In this study, only simulations were conducted, and no experimental studies using actual bamboo rods were performed. The specifications of bamboo rods vary greatly depending on the target fish species and fields. Therefore, experimental verification of the findings of this study will be achieved in future.
